# Supplementary material for: Super-resolution imaging of fluorescent dipoles via polarized structured illumination microscopy
Source: Nat Commun. 2019 Oct 16;10:4694. doi: 10.1038/s41467-019-12681-w (PMC6795901; doi:10.1038/s41467-019-12681-w)
Supplement: Supplementary file 1 — Supplementary Information [file 41467_2019_12681_MOESM1_ESM.pdf]

Supplementary Information

Super-resolution Imaging of the Fluorescent Dipole Assembly with Polarized Structured Illumination Microscopy

Zhanghao et al. Nature Communications. 2019.

Table of Contents

Supplementary Figures 3

Supplementary Figure 1 – Comparison of the observable region of reciprocal space........ 3

Supplementary Figure 2 – Influence of missing spatio-angular cross harmonics........ 4

Supplementary Figure 3 – Diagrams of home-built SLM-SIM system.. 5

Supplementary Figure 4 - The comparison of pSIM performance among the OMX-SIM, the N-SIM, and the SLM-SIM.. 6

Supplementary Figure 5 – The illumination non-uniformity calibration of pSIM. 7

Supplementary Figure 6 - TIRF-pSIM imaging results of Alexa 568 Phalloidin labelled actin filaments. .. 8

Supplementary Figure 7 - Polarization factor comparison between different labelling methods. 9

Supplementary Notes 10

1. Supplementary Note 1 – Structured illumination in spatio-angular hyperspace........ 10
2. Supplementary Note 2 – Details of pSIM reconstruction algorithm........ 12
3. Supplementary Note 3 – Calibration of illumination non-uniformity 14

Supplementary Figures

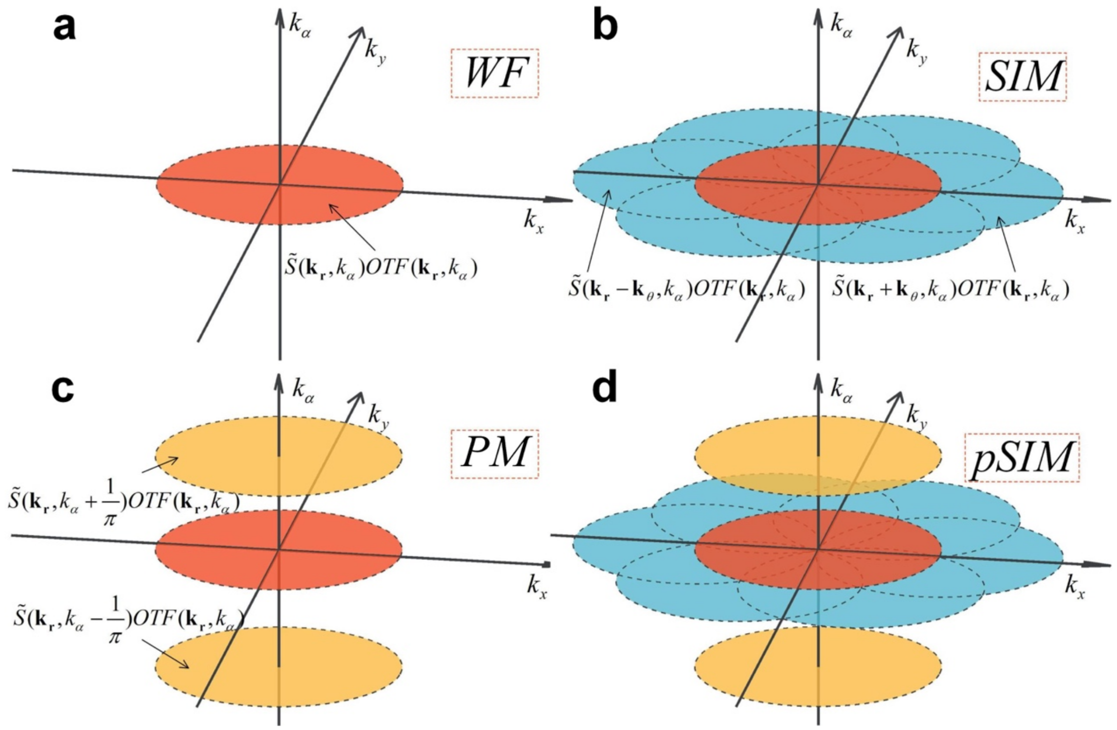

Supplementary Figure 1 – Comparison of the observable region of reciprocal space among wide field (a), SIM (b), PM (c), and pSIM (d). (a) Due to the optical transfer function (OTF) cut-off of a wide-field microscope, the frequency outside the passband is blocked. **(b)** In frequency domain, the product of specimen and modulation frequency becomes convolution, which expands the visible frequency with 6 pedals. **(c)** The polarization modulation also brings two additional angular components in the reciprocal space. **(d)** Combining SIM with polarization modulation leads to super-resolution polarization microscopy, which expands both the spatial and angular observable region.

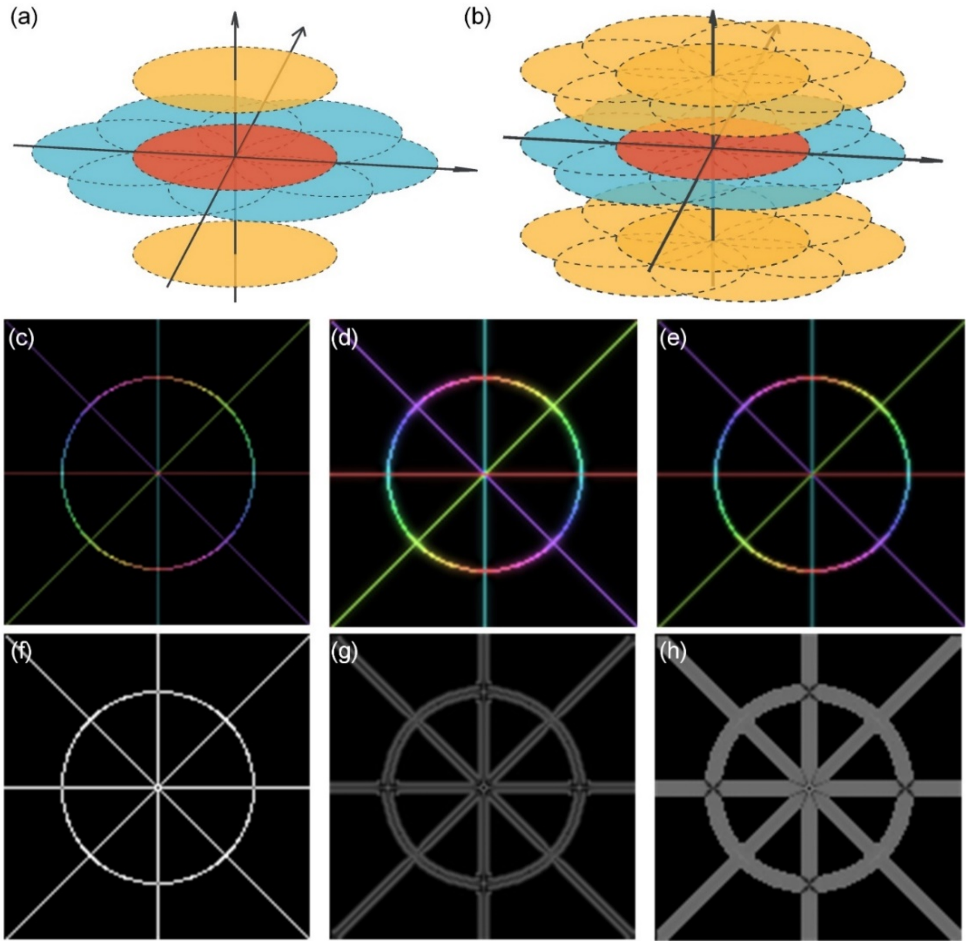

Supplementary Figure 2 – Influence of missing spatio-angular cross harmonics. (a) is the observable region of pSIM in reciprocal space, and **(b)** is the full doubled region with 12 cross harmonics. **(c)** is the simulation setup with radial lines and a circle, with their dipole orientation indicated by pseudocolor. **(d)** is the super-resolution dipole imaging by pSIM using the frequency components in (a). **(e)** is the super-resolution dipole imaging using the frequency components in (b). The results in (d) and (e) are identical to each other. **(f)** is the polarization factor image of the simulated dipoles. **(g)** The missing cross harmonics disrupts the polarization factor image with high-frequency non-uniformity. **(h)** With all harmonics solved, the polarization factor image is consistent to the simulation setup except for the intersection area. By fitting the polarization response with a cosine function (A*cos(2(θ-α)+B) pixel by pixel, θ is the polarization and α is the dipole orientation), the polarization factor is calculated by $\frac{2B}{A+B}$, which equals to the definition elsewhere[1].

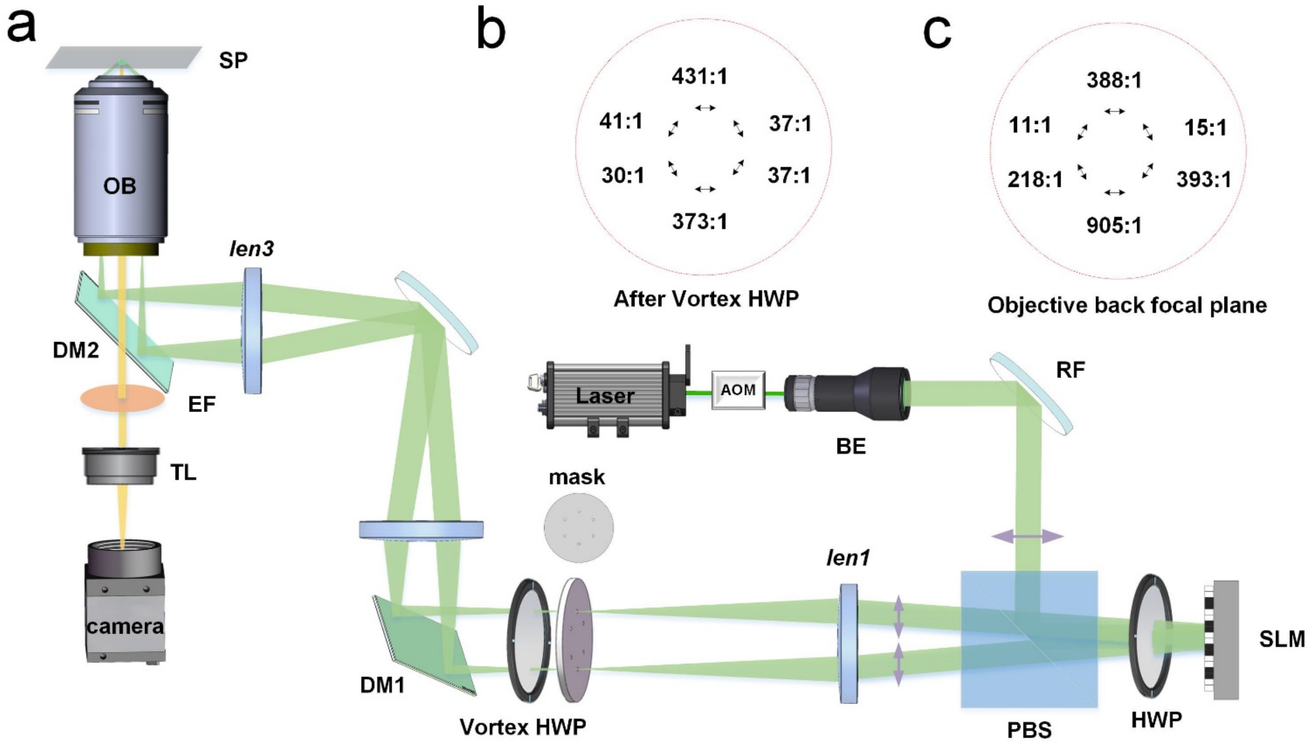

Supplementary Figure 3 – Diagrams of home-built SLM-SIM system. (a) The setup of the SLM-SIM system. AOM: acoustic optical modulator; BE: beam expander; RF: reflecting mirror; PBS: polarization beam splitter; SLM: spatial light modulator; HWP: half-wave plate; DM: dichroic mirror; OB: objective; SP: sample plane; EF: emission filter; TL: tube lens. **(b)** The extinction ratio of s polarization : ps polarization at plane after the vortex HWP. **(c)** The extinction ratio of s polarization : p polarization at the back focal plane of the objective.

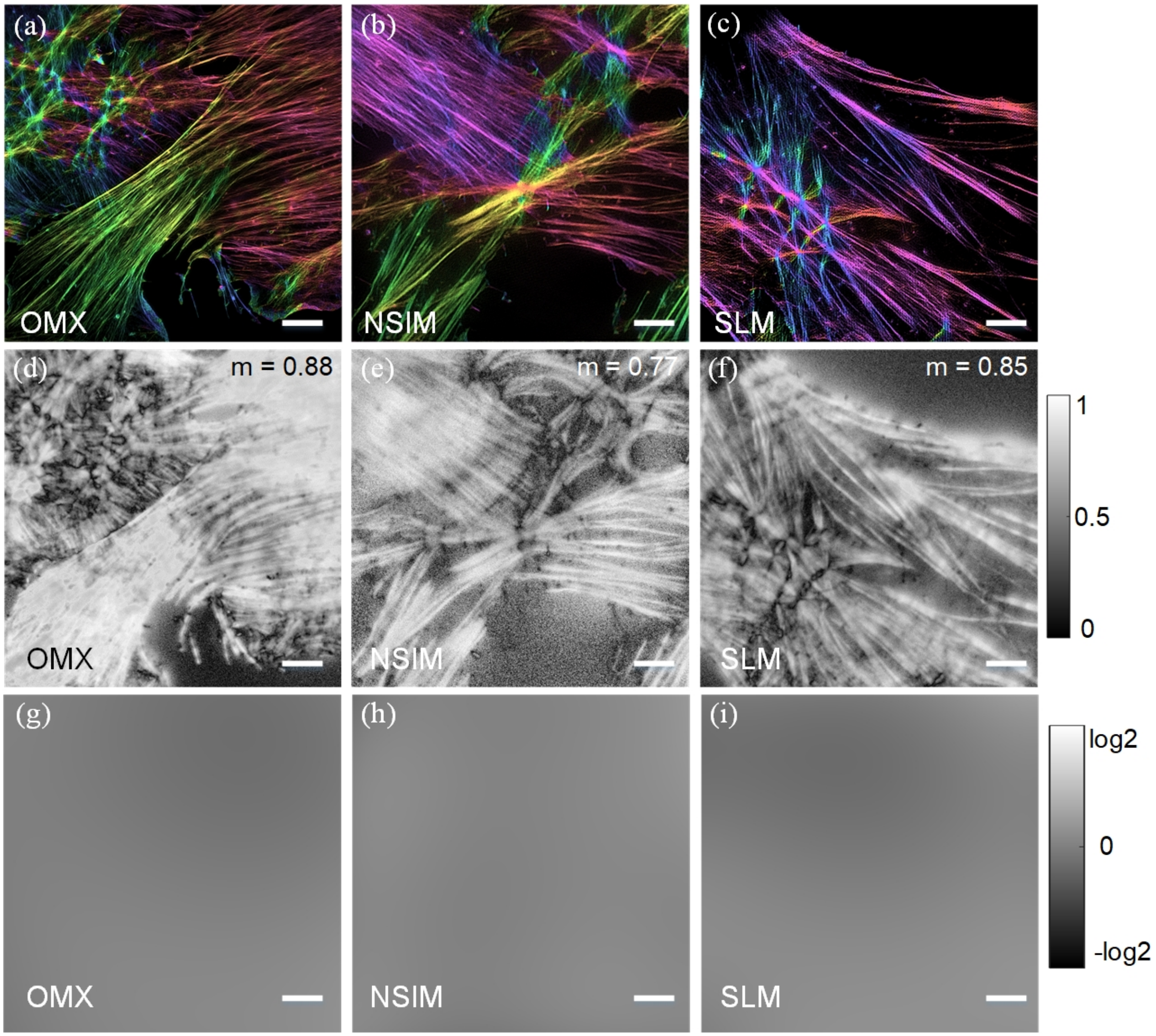

Supplementary Figure 4 - The comparison of pSIM performance among the OMX-SIM, the N-SIM, and the SLM-SIM. (a-c) pSIM imaging results with the three systems of Phalloidin-AF568 labeled actin filaments in U2-OS cells. **(d-f)** The modulation factor (marked at the upper-right corner) calculated by fairSIM[2] represents the contrast of the structured illumination. High modulation factor (close to 1) means the most energy of the lasers beam lies in s polarization. The image of the polarization factor (d-f) represents how the specimen response to the polarization modulation among the three systems. The similar polarization factors among the three images represent that the excitation beams of the three system have a similar degree of polarization. Since the polarization behavior of SLM-SIM is well characterized, the OMX-SIM and the N-SIM system should have similar performance. **(g-i)** The spatial illumination non-uniformity among different illumination patterns of these systems. The illumination intensity ratio of the two patterns ($\log \frac{I_2}{I_1}$) is mapped to the value of the corresponding pixel in the logarithmic form. Scale bar: 5μm.

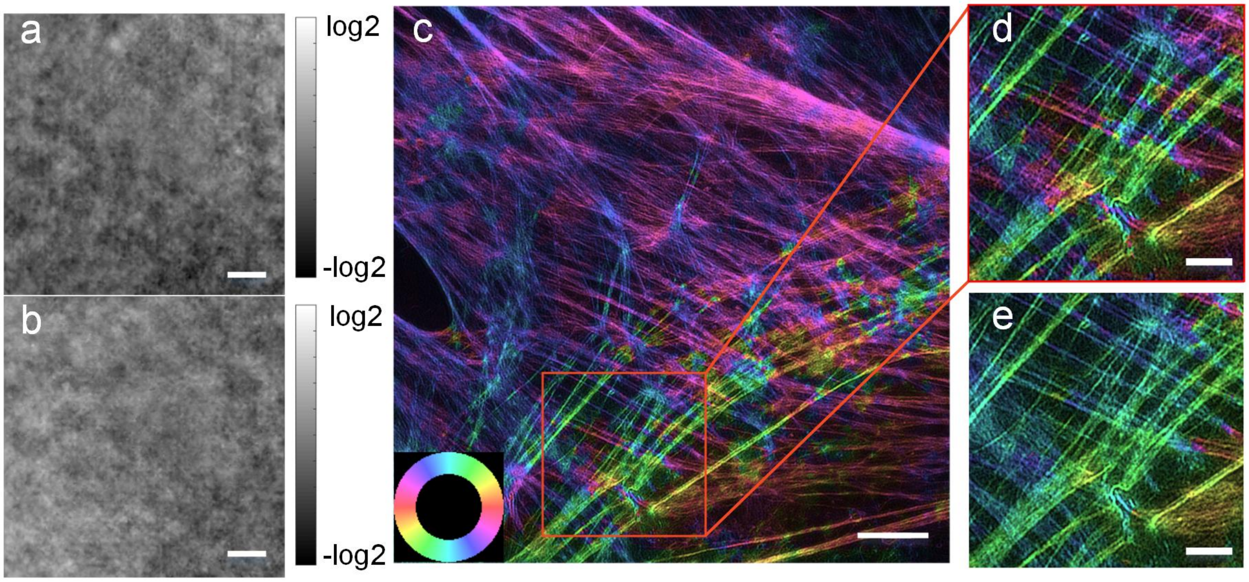

Supplementary Figure 5 – The illumination non-uniformity calibration of pSIM. (a) and **(b)** is the system calibration map of Direction 2 and Direction 3, respectively. The value of each pixel in map represents the illumination intensity ratio of the corresponding direction to Direction 1, displayed in the logarithmic form. **(c)** is the pSIM results with system calibration and zoom in at **(d)**. **(e)** is the pSIM results without the compensation of illumination intensity non-uniformity of the corresponding region in d. The calibration map is generated by imaging the polarization-insensitive fluorescence beads. As the dipole orientation of AF-488 phalloidin labeled actin is parallel to the filament direction, one can obtain a more accurate dipole orientation in (d) over the non-caliberated counterpart (e). The dipole orientation is displayed in pseudo-color indicated by the color wheel. Scale bar: (a-c): 5 µm, (d, e): 2 µm.

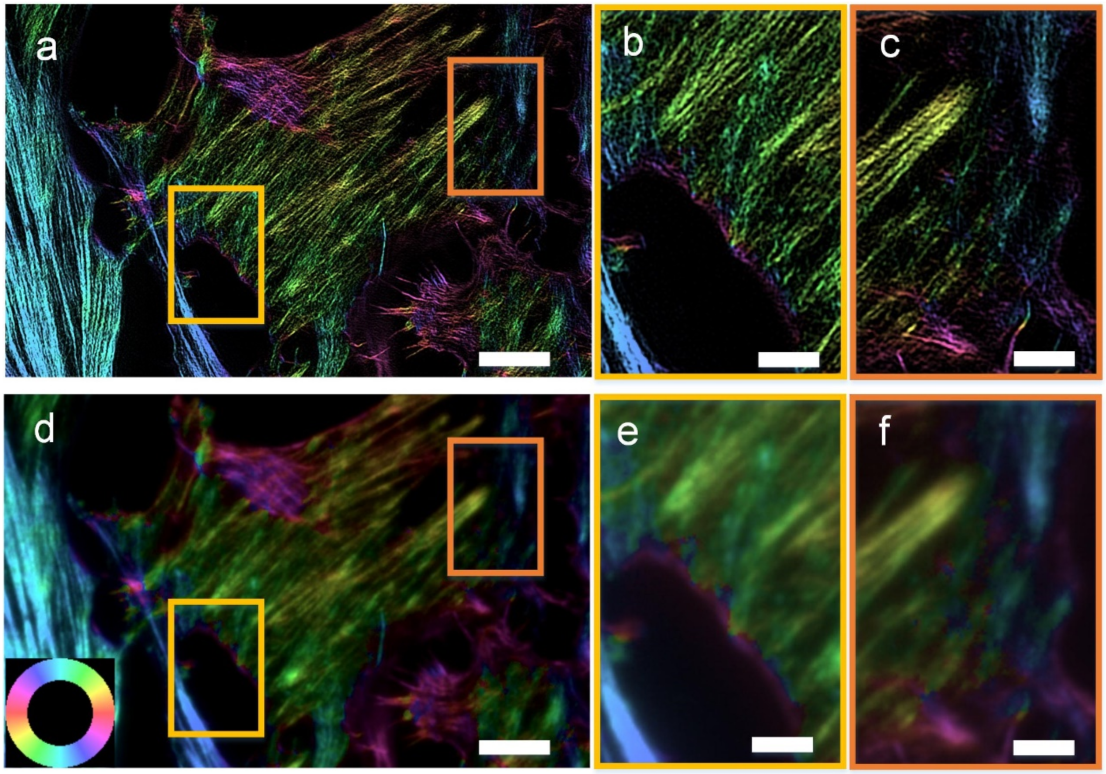

Supplementary Figure 6 - TIRF-pSIM imaging results of Alexa 568 Phalloidin labelled actin filaments. The TIRF-pSIM images are displayed in **(a-c)**, while diffraction-limited TIRF-PM images are compared in **(d-f)**. b (e) and c (f) is the zoom-in view of yellow box and orange box, respectively. Similar to pSIM, TIRF-pSIM is able to measure the in plane dipole orientation since its excitation is also s-polarized. Moreover, because only the specimen close to the coverglass can be excited, the signal to noise ratio can be improved. All the sub-images share the same colorwheel. Scale bar: (a,d) 5μm, (b,c,e,f) 2μm.

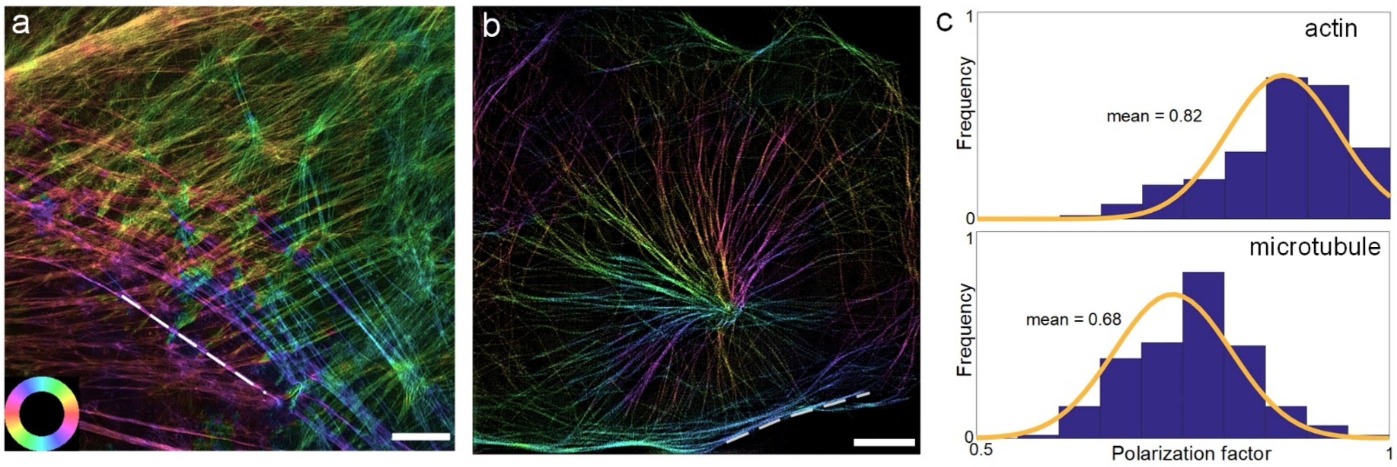

Supplementary Figure 7 - Polarization factor comparison between different labelling methods. (a) pSIM images of phalloidin-AF488 labeled actin filaments in fixed BAPE cells and **(b)** GFP labeled microtubule in live U2OS cells. **(c)** The polarization factor statistics of the regions indicated by the dashed lines. The result shows that the polarization factor of phalloidin-AF488 labeled actin (0.82$\pm$0.08) is higher than GFP labeled microtubule (0.68$\pm$0.09), suggesting that the dipole ensemble of GFPs has a larger wobbling angle. Scale bar: (a) 5 μm, (b) 10 μm.

Supplementary Note 1 - Structured illumination in spatio-angular hyperspace

For the common used wide field microscope, due to the optical transfer function (OTF) cut-off of the objective lens, the high-order frequency information of the sample cannot be detected. Therefore, the observable region is a spatially band-limited region in Fourier domain (Supplementary Figure 1a). In SIM, when a spatial sinusoidal illumination is used to illuminate the specimen, the output signal is their product. The Fourier transform of a sinusoidal signal contains only three components: a DC component and two conjugated peaks. In frequency domain, the product becomes convolution, in which the original frequency is copied three times. The frequency outside the passband is now shifted inside the detectable zone, hence super-resolution can be achieved. SIM uses a spatial sinusoidal pattern $I_{\theta,\varphi}(\mathbf{k_r})$ to illuminate the specimen $S(\mathbf{r})$, which is assumed with isotropic fluorescence polarization. The polarization modulation in SIM is to maintain high-contrast interferometric fringes. Similarly, Polarization Modulation (PM) utilizes the rotational modulation of the excitation polarization direction to measure the dipole orientations, which can be considered as a sinusoidal illumination $F_\theta(\alpha)$ in polarization dimension.

$$\text{SIM: } D_{\theta,\varphi}(\mathbf{r}) = \underbrace{\left[S(\mathbf{r})\cdot I_{\theta,\varphi}(\mathbf{r})\right]}_{Excitation} \otimes PSF(\mathbf{r});\ I_{\theta,\varphi}(\mathbf{r}) = \frac{I_0}{2}\left[1+\cos\left(2\pi\mathbf{p_\theta}\cdot\mathbf{r}+\varphi\right)\right] \qquad \text{Supp. Eq. 1}$$

$$\text{PM: } D_\theta(\mathbf{r};\alpha) = \underbrace{\left[S_p(\mathbf{r},\alpha)\cdot F_\theta(\alpha)\right]}_{Excitation} \otimes PSF(\mathbf{r},\alpha);\ F_\theta(\alpha) = \frac{\eta}{2}\left[1+\cos(2\pi\cdot\frac{1}{\pi}\cdot\alpha-2\theta)\right] \qquad \text{Supp. Eq. 2}$$

Here, $\mathbf{r}$ denotes spatial coordinate and α denotes polarization coordinate. θ is the direction of interferometric fringes or the direction of polarization. In a well-established SIM system, such as the commercial used GE OMX SR and Nikon N-SIM, these two directions are required to be the same for high-contrast patterns. I_0 is the laser power and η is the excitation efficiency. $S(\mathbf{r})$ means polarization-isotropic specimen and $S_p(\mathbf{r},\alpha)$ means polarization-anisotropic specimen. PSF is the point spread function (PSF) of the system. Applying Fourier Transform on Supplementary Eq.2 and Supplementary Eq.3, the observable region of SIM and PM can be represented as (Supplementary Figure 1b, c):

SIM:

$$D_{\theta,\varphi}(\mathbf{k_r}) = \left[S(\mathbf{k_r}) \otimes I_{\theta,\varphi}(\mathbf{k_r})\right] \cdot OTF(\mathbf{k_r});$$

$$I_{\theta,\varphi}(\mathbf{k_r}) = \frac{\pi I_0}{4}\left[\delta(\mathbf{k_r}) + \frac{1}{2}\delta(\mathbf{k_r} - \mathbf{k_\theta})e^{-i\varphi} + \frac{1}{2}\delta(\mathbf{k_r} + \mathbf{k_\theta})e^{i\varphi}\right]$$

Supp. Eq. 3

PM:

$$D_\theta(\mathbf{k_r}, k_\alpha) = \left[S_p(\mathbf{k_r}, k_\alpha) \otimes F_\theta(k_\alpha)\right] \cdot OTF(\mathbf{k_r}, k_\alpha);$$

$$F_\theta(k_\alpha) = \frac{\pi\eta}{4}\left[\delta(k_\alpha) + \frac{1}{2}e^{2i\theta}\delta(k_\alpha - \frac{1}{\pi}) + \frac{1}{2}e^{-2i\theta}\delta(k_\alpha + \frac{1}{\pi})\right]$$

Supp. Eq. 4

Based the similarity between Supplementary Eq.3 and Supplementary Eq.4, it can be conclude that PM is the structured illumination on angular dimension. In SIM system, to generate effective illumination patterns with maximum contrast, polarization modulation is employed to rotate the laser polarization along with the interference direction, so the sample is naturally under the polarized structured illumination. Considering the polarization sensitive sample such as fluorescent dipoles, we can remodel the sample in spatio-angular hyperspace, then the detected images and their Fourier transform would be:

$$D_{\theta,\varphi}(\mathbf{r}, \alpha) = \left[S_p(\mathbf{r}, \alpha) \cdot I_{\theta,\varphi}(\mathbf{r}) \cdot F_\theta(\alpha)\right] \otimes PSF(\mathbf{r}, \alpha)$$

Supp. Eq. 1

$$D_{\theta,\varphi}(\mathbf{k_r}, k_\alpha) = \left[S_p(\mathbf{k_r}, k_\alpha) \otimes I_{\theta,\varphi}(\mathbf{k_r}) \otimes F_\theta(k_\alpha)\right] \cdot OTF(\mathbf{k_r}, k_\alpha)$$

Supp. Eq. 2

The spatial structured illumination term $I_{\theta,\varphi}(\mathbf{r})$ and polarization structured illumination term $F_\theta(\alpha)$ brings larger observable region on spatial dimensions and polarization dimension, respectively. pSIM could enlarge the observable region reciprocal space (Supplementary Figure 1d), which would enable the super-resolution imaging of fluorescent dipoles.

Supplementary Note 2 - Details of pSIM reconstruction algorithm

The expanded form of Eq.6 is:

$$
\begin{aligned}
D_{\theta,\varphi}(\mathbf{k_r},k_\alpha) &= \left[S_p(\mathbf{k_r},k_\alpha)\otimes \tilde{I}_{\theta,\varphi}(\mathbf{k_r})\otimes \tilde{F}_\theta(k_\alpha)\right]\cdot OTF(\mathbf{k_r},k_\alpha) \\
&= \frac{\eta I_0}{4}\left\{ \begin{matrix} S_p(\mathbf{k_r},k_\alpha)\otimes\left[\delta(\mathbf{k_r})+\frac{1}{2}\delta(\mathbf{k_r}-\mathbf{k_\theta})e^{i\varphi}+\frac{1}{2}\delta(\mathbf{k_r}+\mathbf{k_\theta})e^{-i\varphi}\right] \\ \otimes\left[\delta(k_\alpha)+\frac{1}{2}\delta(k_\alpha-\frac{1}{\pi})e^{-i2\theta}+\frac{1}{2}\delta(k_\alpha+\frac{1}{\pi})e^{i2\theta}\right] \end{matrix}\right\}\cdot OTF(\mathbf{k_r},k_\alpha) \\
&= \frac{\eta I_0}{4}\left\{ \begin{matrix} S_p(\mathbf{k_r},k_\alpha)+\frac{1}{2}S_p(\mathbf{k_r},k_\alpha-\frac{1}{\pi})e^{-i2\theta}+\frac{1}{2}S_p(\mathbf{k_r},k_\alpha+\frac{1}{\pi})e^{i2\theta} \\ +e^{i\varphi}\left[S_p(\mathbf{k_r}-\mathbf{k_\theta},k_\alpha)+\frac{1}{2}S_p(\mathbf{k_r}-\mathbf{k_\theta},k_\alpha-\frac{1}{\pi})e^{-i2\theta}+\frac{1}{2}S_p(\mathbf{k_r}-\mathbf{k_\theta},k_\alpha+\frac{1}{\pi})e^{i2\theta}\right] \\ +e^{-i\varphi}\left[S_p(\mathbf{k_r}+\mathbf{k_\theta},k_\alpha)+\frac{1}{2}S_p(\mathbf{k_r}+\mathbf{k_\theta},k_\alpha-\frac{1}{\pi})e^{-i2\theta}+\frac{1}{2}S_p(\mathbf{k_r}+\mathbf{k_\theta},k_\alpha+\frac{1}{\pi})e^{i2\theta}\right] \end{matrix}\right\}\cdot OTF(\mathbf{k_r},k_\alpha)
\end{aligned}
\quad \text{Eq. 7}
$$

Given a specific θ_i, there are 9 unknown variables in Eq.6: $\tilde{S}_p(\mathbf{k_r},k_\alpha)$, $\tilde{S}_p(\mathbf{k_r},k_\alpha \pm \frac{1}{\pi})$, $\tilde{S}_p(\mathbf{k_r}\pm\mathbf{k_{\theta_i}},k_\alpha)$ $\tilde{S}_p(\mathbf{k_r}\pm\mathbf{k_{\theta_i}},k_\alpha\pm\frac{1}{\pi})$. So the coefficient matrix is singular, the linear equation cannot be resolved. We set:

$$
\begin{aligned}
\tilde{S}_{p0\theta_i} &= S_p(\mathbf{k_r},k_\alpha)+\frac{1}{2}S_p(\mathbf{k_r},k_\alpha-\frac{1}{\pi})e^{-i2\theta_i}+\frac{1}{2}S_p(\mathbf{k_r},k_\alpha+\frac{1}{\pi})e^{i2\theta_i} \\
\tilde{S}_{p1\theta_i} &= S_p(\mathbf{k_r}-\mathbf{k_{\theta_i}},k_\alpha)+\frac{1}{2}S_p(\mathbf{k_r}-\mathbf{k_{\theta_i}},k_\alpha-\frac{1}{\pi})e^{-i2\theta_i}+\frac{1}{2}S_p(\mathbf{k_r}-\mathbf{k_{\theta_i}},k_\alpha+\frac{1}{\pi})e^{i2\theta_i} \\
\tilde{S}_{p2\theta_i} &= S_p(\mathbf{k_r}+\mathbf{k_{\theta_i}},k_\alpha)+\frac{1}{2}S_p(\mathbf{k_r}+\mathbf{k_{\theta_i}},k_\alpha-\frac{1}{\pi})e^{-i2\theta_i}+\frac{1}{2}S_p(\mathbf{k_r}+\mathbf{k_{\theta_i}},k_\alpha+\frac{1}{\pi})e^{i2\theta_i}
\end{aligned}
\quad \text{Eq. 3}
$$

With the three inputs $\varphi_1,\varphi_2,\varphi_3$, we can calculate these three units $\tilde{S}_{p0\theta_i},\tilde{S}_{p1\theta_i},\tilde{S}_{p2\theta_i}$ by solving the following linear equations:

$$
\begin{bmatrix} D_{\theta_i,\varphi_1}(\mathbf{k_r},k_\alpha) \\ D_{\theta_i,\varphi_2}(\mathbf{k_r},k_\alpha) \\ D_{\theta_i,\varphi_3}(\mathbf{k_r},k_\alpha) \end{bmatrix} = M_{SIM}\cdot \begin{bmatrix} \tilde{S}_{p0\theta_i}\cdot OTF(\mathbf{k_r},k_\alpha) \\ \tilde{S}_{p1\theta_i}\cdot OTF(\mathbf{k_r},k_\alpha) \\ \tilde{S}_{p2\theta_i}\cdot OTF(\mathbf{k_r},k_\alpha) \end{bmatrix}; M_{SIM} = \frac{\eta I_0}{4}\begin{bmatrix} 1 & \frac{1}{2}e^{i\varphi_1} & \frac{1}{2}e^{-i\varphi_1} \\ 1 & \frac{1}{2}e^{i\varphi_2} & \frac{1}{2}e^{-i\varphi_2} \\ 1 & \frac{1}{2}e^{i\varphi_3} & \frac{1}{2}e^{-i\varphi_3} \end{bmatrix} \quad \text{Eq. 4}
$$

With three pattern directions $\theta_1, \theta_2, \theta_3$, we should notice that $\tilde{S}_{p0\theta_1}, \tilde{S}_{p0\theta_2}, \tilde{S}_{p0\theta_3}$ is the linear combination of the same components $S_p(\mathbf{k_r}, k_\alpha), S_p(\mathbf{k_r}, k_\alpha - \frac{1}{\pi}), S_p(\mathbf{k_r}, k_\alpha + \frac{1}{\pi})$ with different coefficients. Therefore, they can be calculated by solving following linear equations:

$$\begin{bmatrix} \tilde{S}_{p0\theta_i} \\ \tilde{S}_{p1\theta_i} \\ \tilde{S}_{p2\theta_i} \end{bmatrix} = M_{LD} \cdot \begin{bmatrix} \tilde{S}_p(\mathbf{k_r}, k_\alpha) \\ \tilde{S}_p(\mathbf{k_r}, k_\alpha - \frac{1}{\pi}) \\ \tilde{S}_p(\mathbf{k_r}, k_\alpha + \frac{1}{\pi}) \end{bmatrix}; M_{LD} = \begin{bmatrix} 1 & \frac{1}{2}e^{-i2\theta_1} & \frac{1}{2}e^{i2\theta_1} \\ 1 & \frac{1}{2}e^{-i2\theta_2} & \frac{1}{2}e^{i2\theta_2} \\ 1 & \frac{1}{2}e^{-i2\theta_3} & \frac{1}{2}e^{i2\theta_3} \end{bmatrix} \quad \text{Eq. 5}$$

As the ±1 harmonic on angular axis $\tilde{S}_p(\mathbf{k_r}, k_\alpha \pm \frac{1}{\pi})$ can be resolved, the dipole orientation could be obtained from the phase of +1 harmonic.

It should be noticed that the six spatio-angular cross harmonics $\tilde{S}_p(\mathbf{k_r} \pm \mathbf{k}_{\theta_i}, k_\alpha \pm \frac{1}{\pi})$ are unsolvable based on Supplementary Eq. 8, because excitation polarization θ and illumination vector $\mathbf{k_\theta}$ are dependent on each other. We build a simulation model to verify that these missing harmonics do not influence either spatial image or dipole orientation image (Supplementary Figure 2).

Supplementary Note 3 - Calibration of illumination non-uniformity

In pSIM setup, since the polarization of excitation implemented by rotating the interferometric pattern, the illumination intensity may fluctuate among different polarizations. This non-uniformity will significantly affect the measurement of dipole orientation. To solve this problem, we use the specimen of sparse distributed 100-nm fluorescent beads, which emitted fluorescence signal is insensitive to polarization excitation, to calibrate the intensity non-uniformity of our system. Since for 2D SIM, 3 illumination patterns are implemented, we use two spatial calibration maps to demonstrate the intensity non-uniformity of Pattern 2 referring to Pattern 1, and Pattern 3 referring to Pattern 1, respectively. The generation of these calibration maps takes the following steps:

1) We move the specimen with a micro-stage at a step size of 500 nm. At each position, we capture a SIM image sequence. For 2D SIM, there should be 9 images in a sequence corresponding to three illumination patterns multiply three phases in each direction.

2) At each position, we obtain three wide field images corresponding to three illumination patterns. For each illumination direction, the wide field image is generated by average the images of three phases, since phase different is designed to be 2π/3.

3) For each wide field image, we run quickPALM (http://code.google.com/p/quickpalm)[3] to localize the beads and obtain their positions and intensities.

4) For each bead appears on all three images, we calculate ratio of its intensity in Pattern 2 and Pattern 3 to its intensity in Pattern 1, respectively. The ratio reflects the illumination non-uniformity among different patterns at the location of the beads. We set this ratio to the corresponding location in the calibration map.

5) By moving the specimen and repeat Step 2 to Step 4, we can get enough measurements. The calibration map of the whole field-of-view can be generated by interpolation. (Supplementary Figure 5 a, b)

Using the generated calibration map, we can compensate the intensity non-uniformity in the "PM step" during the pSIM reconstruction. Considering intensity non-uniformity, the detected image and its Fourier transformation can be represented as:

$$D(\mathbf{r}) = (S(\mathbf{r})\ M(\mathbf{r})\ I(\mathbf{r})) \otimes PSF(\mathbf{r}),$$

$$\tilde{D}(\mathbf{k_r}) = (\tilde{S}(\mathbf{k_r}) \otimes \tilde{M}(\mathbf{k_r}) \otimes \tilde{I}(\mathbf{k_r}))\ OTF(\mathbf{k_r}),$$

Eq. 12

where M denotes the non-uniformity, S denotes the sample, I denotes the illumination, PSF denotes the system point spread function and OTF denotes system optical transfer function.

Based on Supplementary Eq. 12, the compensation of intensity non-uniformity takes the following steps:

1) Apply Fourier transform to the detected images
2) Divide by the OTF in Fourier domain and apply inverse Fourier transform.
3) Divide by the calibration map of the corresponding illumination pattern in spatial domain
4) Apply Fourier transform and multiply OTF in Fourier domain

After these steps, the system non-uniformity calibrated images can be obtained. The comparison of pSIM reconstruction with and without calibration is shown in Supplementary Figure 5. The illumination non-uniformity calibration has been embedded in pSIM reconstruction.

References:

1. Mehta, S.B. *et al.* Dissection of molecular assembly dynamics by tracking orientation and position of single molecules in live cells. *Proceedings of the National Academy of Sciences* **113**, E6352-E6361 (2016).
2. Muller, M., Monkemoller, V., Hennig, S., Hubner, W. & Huser, T. Open-source image reconstruction of super-resolution structured illumination microscopy data in ImageJ. *Nat Commun* **7**, 10980 (2016).
3. Henriques, R. *et al.* QuickPALM: 3D real-time photoactivation nanoscopy image processing in ImageJ. *Nature methods* **7**, 339 (2010).
