# Supplementary material for: Super-resolution imaging of fluorescent dipoles via polarized structured illumination microscopy
Source: Nat Commun. 2019 Oct 16;10:4694. doi: 10.1038/s41467-019-12681-w (PMC6795901; doi:10.1038/s41467-019-12681-w)
Supplement: Supplementary file 3 — Description of Additional Supplementary Files [file 41467_2019_12681_MOESM3_ESM.pdf]

Description of Additional Supplementary Files

File name: Supplementary Movie 1
Description: PM images of Alexa 488 phalloidin labeled actin filaments in BPAE cells. Scale bar: 2 μm.

File name: Supplementary Movie 2
Description: Volumetric display of the 3D distribution of the actin filaments in the mouse kidney tissue slice. The 3D-SIM raw images were taken with GE DeltaVision OMX SR. The 3D volume was rendered with Imaris (http://www.bitplane.com/).

File name: Supplementary Movie 3
Description: Time elapse imaging of the myosin-driven movement of Phalloidin-Alexa 488 labeled actins. Scale bar: 5 μm.

File name: Supplementary Movie 4
Description: Time elapse imaging of a fragment of Phalloidin-Alexa 488 labeled actins. Scale bar:500 nm.

File name: Supplementary Movie 5
Description: Live cell imaging of GFP labelled microtubule in U2-OS cells. Scale bar: 1 μm.
